# Nasal Chondromesenchymal Hamartoma: Rare Case Report in an Elderly Patient and Brief Review of Literature

**DOI:** 10.1155/2018/5971786

**Published:** 2018-10-14

**Authors:** Kanish Mirchia, Rana Naous

**Affiliations:** Department of Pathology, SUNY Upstate Medical University, Syracuse, NY 13210, USA

## Abstract

Hamartomas are considered a mixture of nonneoplastic tissue, which may be indigenous to a different location in the body. As such, they may be epithelial, mesenchymal, or mixed. In the sinonasal region, the following hamartomatous lesions are considered to lie on a spectrum and include respiratory epithelial adenomatoid hamartoma (REAH), chondro-osseous respiratory epithelial adenomatoid hamartoma (COREAH), and nasal chondromesenchymal hamartoma (NCMH). To our knowledge, less than 50 cases of sinonasal hamartomas have been reported in the English literature so far with NCMH being very rare and primarily a tumor in infancy, with only 2 cases reported in individuals older than 16 years of age. We report a highly unusual case of a NCMH in the right maxillary sinus of a 70-year-old female.

## 1. Case Report

A 70-year-old female presented with a two-year history of slowly growing, nonpainful maxillary sinus mass. She has a history of chronic maxillary sinusitis corresponding to presentation of the mass, with the first episode reported in 2014. Computed tomography (CT) imaging revealed an erosive right maxillary sinus mass (2.5 x 2.1 cm) with bony destruction.

**Figure 1:**
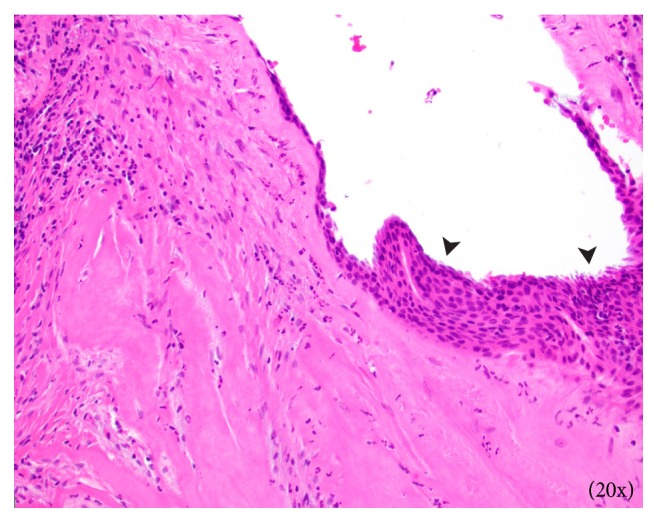
Area of respiratory lining epithelium with squamous metaplastic change (arrowheads).

**Figure 2:**
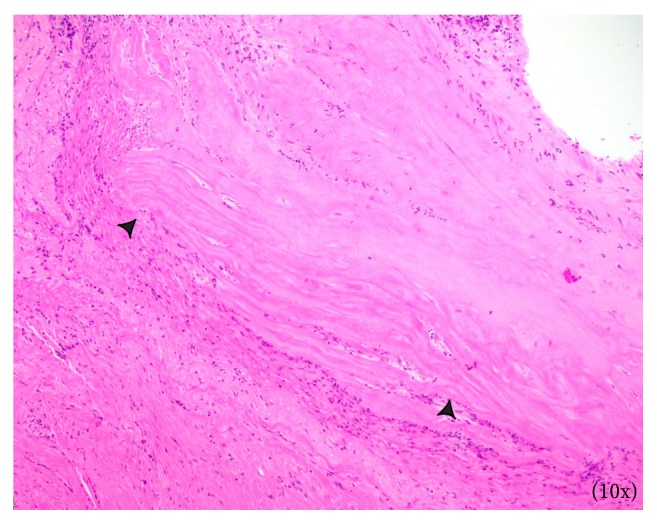
Focal areas of stroma displaying hyalinization (arrowheads).

**Figure 3:**
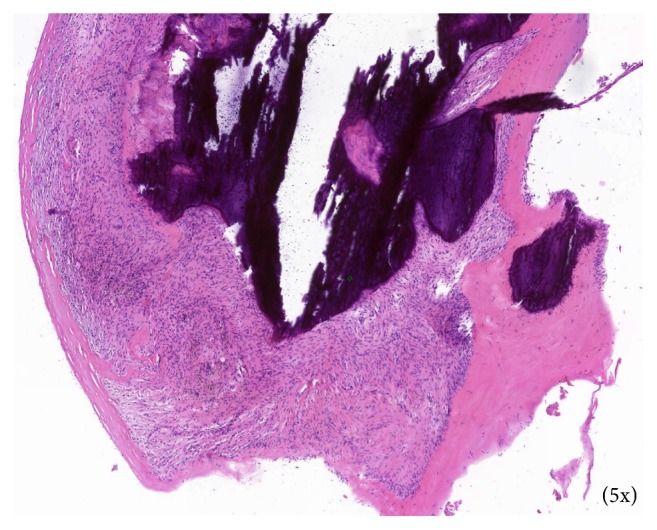
Focal areas displaying calcification and ossification surrounded by variably spindled stroma.

**Figure 4:**
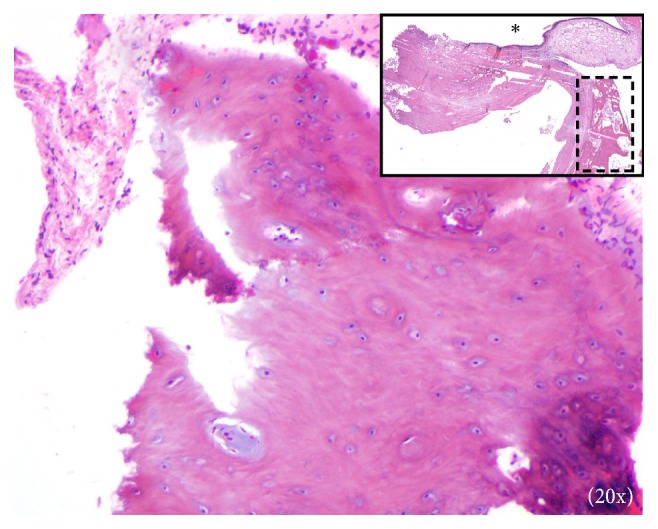
Chondroid regions which support the hamartomatous nature of the lesion. Inset shows area at low-power with spatial relation of components, including surface ciliated epithelium (*∗*) and bone (within dashed lines).

**Figure 5:**
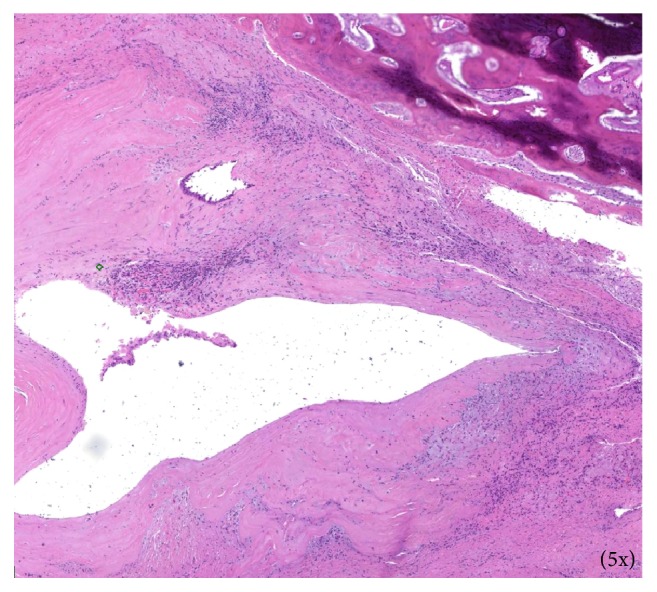
Variably dilated cystic regions within the lesion.

**Figure 6:**
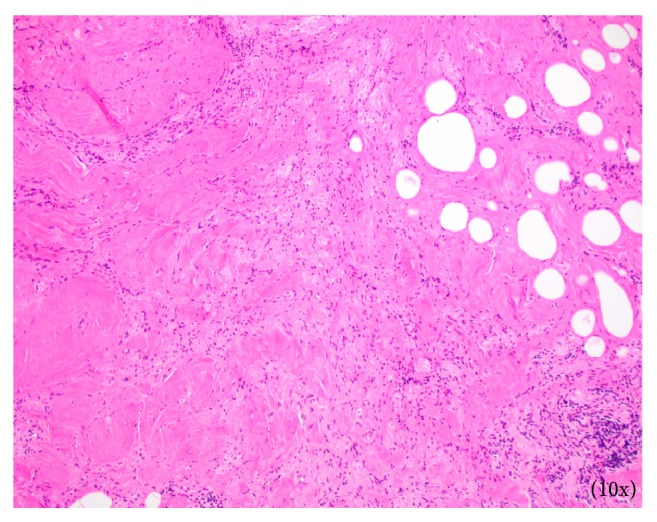
Disorganized bundles of nervous tissue interspersed within collagenous stroma.

**Figure 7:**
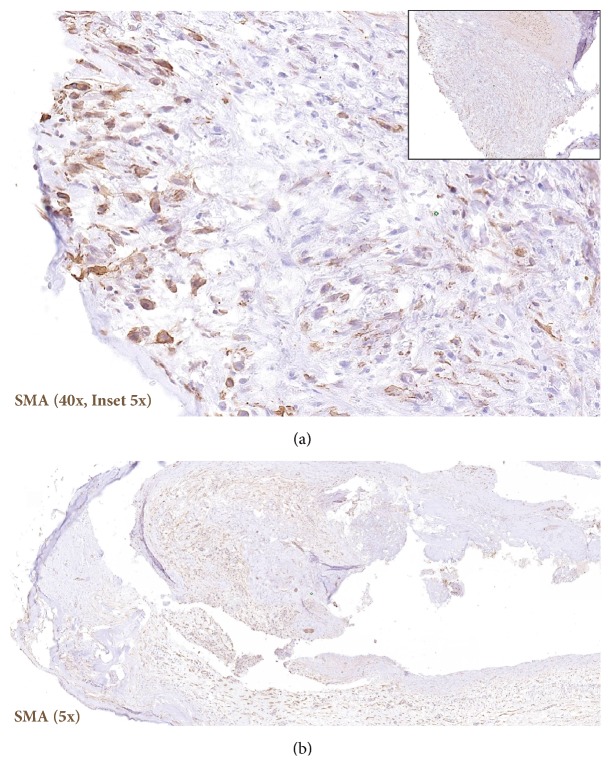
Immunohistochemistry for smooth muscle actin (SMA) showing positive staining in the spindled lesional cells.

**Figure 8:**
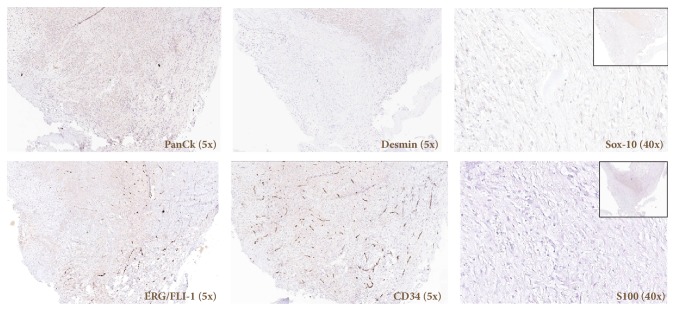
Lesional cells are negative for pan-cytokeratin (ae1/ae3), desmin, Sox-10, and S100. CD34 and ERG/FLI-1 highlight vascular endothelial cells.

Surgical excision of the right maxillary sinus mass revealed a fragmented, white, vaguely nodular, and whorled lesion. Histological examination revealed fragments of respiratory-type epithelium with focal cystic invagination and associated squamous metaplasia [Figure 1]. The underlying stroma consisted of a variably cellular, benign spindle cell proliferation with an associated background of hyalinization [Figure 2], calcification and ossification [Figure 3], and focal chondroid change [Figure 4] in a vague lobule-like arrangement. Focal areas of aneurysmal and cystic changes [Figure 5] were seen which would provide an explanation for the clinically noted enlargement since hamartomas by definition would be expected to have a much lower rate of growth. The intrinsic slow-growing nature is also supported by the deficit of mitotic activity even in the highly cellular/spindled regions of the lesion (less than 1/10 hpf). Areas with haphazard arrangement of nerve bundles within the collagenous stroma [Figure 6] were also noted. Immunohistochemical stains were positive for SMA [Figures 7(a) and 7(b)] in the spindle cells and negative for CK AE1/AE3, EMA, CD34, Stat6, ERG/FLI-1, Mucin 4, S-100, Sox-10, and desmin [Figure 8]; ruling out perineurioma, solitary fibrous tumor, a vascular neoplasm, Evans tumor, a benign peripheral nerve sheath tumor, or a myogenic neoplasm. The overall findings were suggestive of a hamartomatous lesion, most likely a nasal chondromesenchymal hamartoma. The absence of submucosal glandular proliferation, myxoid stroma, or mucinous metaplasia in the lining epithelium lowers the likelihood of other neoplastic hamartomatous lesions such as COREAH.

## 2. Discussion

Nasal chondromesenchymal hamartomas are most commonly seen in the nasal cavity of children less than 3 months old, with less common involvement of the paranasal sinuses [2]. As per one review [1], mean age for NCMH was 9.6 years. Review of the English PubMed literature reveals 43 cases [Table 1] of NCMH previously published, with our case being the oldest patient reported, and presenting with a tumor in an unusual location.

Our case would lend support to extending the age range for NCMH and considering it in the differential diagnosis of all sinonasal region tumors, irrespective of age, and location in the head and neck region. Despite primarily being a benign lesion, these tumors can present with areas of necrosis and local destruction, including bony invasion. The tumors can be aggressive appearing on imaging, extending into bony structures, including the cranium and/or the orbital cavity, which should not lead away from the diagnosis of this benign lesion. Detailed CT or preferably MRI prior to surgical excision should be performed.

NCMH has been associated with development of pleuropulmonary blastoma (PPB) during infancy. A recent [28] report highlighted the association of NCMH and PPB with DICER1 mutation and various associated entities such as lung cysts, cystic nephroma, renal sarcoma, Wilms tumor, thyroid hyperplasia, and CNS tumors. NCMH in isolation however is a benign lesion with follow-up in patients up to 16 years after excision, except for one reported case with malignant transformation in the literature [20]. Etiologically, it would make sense that cases in adults, such as ours, represent a tissue response to insult, such as chronic sinusitis rather than an inborn germline error (such as a DICER1 mutation).

Whether the presentation of a NCMH at a later age predisposes to malignant transformation due to the long-standing nature of the lesion is up for debate. It could represent a somatic DICER1 mutation rather than a germline mutation, causing the hamartoma to form later in age. Longer follow-up results from the adult cases and routine genetic testing in all NCMH will help provide an answer to these questions.

## 3. Conclusion

We report an unusual case of NCMH eroding the right maxillary sinus of a 70-year-old female. Although, NCMH is a rare entity with predilection for pediatric age groups, it is important to consider NCMH in the differential diagnosis of nasal/sinonasal masses in adult patients in order to avoid diagnostic errors.

## Figures and Tables

**Table 1:** Brief review of cases of nasal chondromesenchymal hamartomas reported in the English literature. Some cases also reported in older review articles [1].

Age	Sex	Follow-up (Asymptomatic)	Site	Pertinent Information	Study	Year
5 days	M	2 years	Nasal cavity	-	[2] McDermott	1998

12 days	F	< 16 months	Nasal cavity	Intracranial extension	[2] McDermott	1998

14 days	M	-	Nasal cavity Ethmoid Sinus	Intracranial extension Residual tumor	[2] McDermott	1998

2 months	M	18 months	Nasal cavity	Intracranial extension	[2] McDermott	1998

3 months	F	2 years	Nasal cavity Ethmoid Sinus	Intracranial extension Residual tumor	[2] McDermott	1998

3 months	M	4 years	Nasal cavity	-	[2] McDermott	1998

***7 years***	*M*	*-*	*Nasal cavity Sphenoid sinus*	***PPB*** *, multiple recurrences*	*[2] McDermott*	*1998*

4 months	M	13 years	Nasal cavity	Intracranial extension	[3] Kato	1999

0 days	M	5 years	Nasal cavity Sphenoiod sinus Ethmoid sinus	Orbital compression	[4] Hsueh	2001

9 months	M	9 months	Nasal cavity	-	[4] Hsueh	2001

*16 years*	*M*	*8 months*	*Nasal cavity*	*3-month history*	*[5] Alrawi M*	*2003*

5 months	M	-	Nasal cavity	Orbital compression	[6] Kim B	2004

*11 years*	*M*	*-*	*Nasal cavity Ethmoid sinus*	*8-month history*	*[7] Norman ES*	*2004*

1 year	M	-	Nasal cavity	Orbital extension Residual tumor	[8] Shet T	2004

*11 years*	*M*	*-*	*Nasal cavity Ethmoid sinus*	*-*	*[9] Ozolek JA*	*2005*

*17 years*	*F*	*-*	*Nasal cavity*	*-*	*[9] Ozolek JA*	*2005*

*25 years*	*M*	*-*	*Nasal cavity Maxillary sinus*	***Bilateral*** * NCMH* *Intracranial aneurysms*	*[9] Ozolek JA*	*2005*

*69 years*	*F*	*-*	*Nasal cavity Ethmoid sinus*	*-*	*[9] Ozolek JA*	*2005*

*11 years*	*M*	*2 months*	*Nasal cavity*	*-*	*[10] Low SE*	*2006*

***15 years***	*F*	*6 months*	*Nasal cavity*	***Bilateral*** * NCMH* ***PPB***	*[11] Johnson C*	*2007*

7 months	M	18 months	Nasal cavity	Orbital compression	[12] Silkiss RZ	2007

12 months	M	-	Nasal cavity	Orbital compression	[13] Finitsis S	2009

19 months	M	10 months	Nasal cavity	Intracranial, orbital extension	[14] Kim JE	2009

***2 cases previously reported, both with PPB, multiple recurrences***					[15] Priest JR	2010

*10 years*	*F*	*21 months*	*Nasal cavity*	***Bilateral*** * NCMH* ***PPB***	*[15] Priest JR*	*2010*

*11 years*	*M*	*4 months*	*Nasal cavity*	***PPB***	*[15] Priest JR*	*2010*

*11 years*	*M*	*-*	*-*	***PPB***	*[16] Behery RE*	*2012*

*8 years*	*M*	*6 months*	*Sphenoid sinus Ethmoid sinus*	*4-month history*	*[17] Uzomefuna*	*2012*

*14 years*	*M*	*4 years*	*Nasal cavity Maxillary sinus*	*-*	*[18] Cho YC*	*2013*

*23 years*	*M*	*3 months*	*Nasal cavity Ethmoid Sinus*	*Orbital extension*	*[19] Li GY*	*2013*

*40 years*	*F*	*-*	*Nasal cavity Ethmoid Sinus* *Maxillary sinus*	***Malignant transformation Recurrence***	*[20] Li Y*	*2013*

9 months	F	-	Nasal cavity Maxillary sinus	Orbital compression	[21] Moon S	2014

*14 years*	*M*	*-*	*Nasal cavity*	***Bilateral*** * NCMH* ***PPB***	*[22] Obidan AA*	*2014*

6 weeks	F	10 months	Nasal cavity	-	[23] Wang T	2014

*5 years*	*M*	*3 years*	*Nasal cavity* *Ethmoid sinus*	*4-year history*	*[23] Wang T*	*2014*

10 months	M	18 months	Nasal cavity	6-month history	[24] Lee CH	2015

*49 years*	*M*	*2 years* *4 years (phone)*	*Nasal cavity*	*5-year history*	*[1] Mason AK*	*2015*

Systematic review					[1] Mason AK	2015

*5 years*	*M*	*-*	*Nasal cavity*	*Previous rhabdomyosarcoma in remission*	*[25] Avci H*	*2016*

*13 years*	*F*	*12 months*	*Nasal cavity*	*6-month history*	*[26] Unal A*	*2016*

3 years	M	3 years	Nasal cavity	-	[27] Nakaya M	2017

*Index*. *Cases older than 1 year of age at presentation*. ***Bilateral/cases associated with pleuropulmonary blastoma***.
